# Evaluation of Two Lyophilized Molecular Assays to Rapidly Detect Foot‐and‐Mouth Disease Virus Directly from Clinical Samples in Field Settings

**DOI:** 10.1111/tbed.12451

**Published:** 2015-11-30

**Authors:** E. L. A. Howson, B. Armson, M. Madi, C. J. Kasanga, S. Kandusi, R. Sallu, E. Chepkwony, A. Siddle, P. Martin, J. Wood, V. Mioulet, D. P. King, T. Lembo, S. Cleaveland, V. L. Fowler

**Affiliations:** ^1^ The Pirbright InstitutePirbrightSurreyUK; ^2^ Institute of Biodiversity, Animal Health and Comparative MedicineCollege of Medical, Veterinary & Life SciencesGraham Kerr BuildingUniversity of GlasgowGlasgowUK; ^3^ Faculty of Veterinary MedicineSokoine University of AgricultureMorogoroTanzania; ^4^ Tanzania Veterinary Laboratory AgencyDar‐es‐SalaamTanzania; ^5^ Foot‐and‐Mouth Disease LaboratoryMinistry of Agriculture, Livestock and FisheriesNairobiEmbakasiKenya; ^6^ OptiGene LimitedHorshamWest SussexSalisburyUK; ^7^ Enigma Diagnostics LimitedSalisburyWiltshireUK

**Keywords:** foot‐and‐Mouth disease, foot‐and‐mouth disease virus, diagnostics, rRT‐PCR, RT‐LAMP, lyophilized

## Abstract

Accurate, timely diagnosis is essential for the control, monitoring and eradication of foot‐and‐mouth disease (FMD). Clinical samples from suspect cases are normally tested at reference laboratories. However, transport of samples to these centralized facilities can be a lengthy process that can impose delays on critical decision making. These concerns have motivated work to evaluate simple‐to‐use technologies, including molecular‐based diagnostic platforms, that can be deployed closer to suspect cases of FMD. In this context, FMD virus (FMDV)‐specific reverse transcription loop‐mediated isothermal amplification (RT‐LAMP) and real‐time RT‐PCR (rRT‐PCR) assays, compatible with simple sample preparation methods and *in situ* visualization, have been developed which share equivalent analytical sensitivity with laboratory‐based rRT‐PCR. However, the lack of robust ‘ready‐to‐use kits’ that utilize stabilized reagents limits the deployment of these tests into field settings. To address this gap, this study describes the performance of lyophilized rRT‐PCR and RT‐LAMP assays to detect FMDV. Both of these assays are compatible with the use of fluorescence to monitor amplification in real‐time, and for the RT‐LAMP assays end point detection could also be achieved using molecular lateral flow devices. Lyophilization of reagents did not adversely affect the performance of the assays. Importantly, when these assays were deployed into challenging laboratory and field settings within East Africa they proved to be reliable in their ability to detect FMDV in a range of clinical samples from acutely infected as well as convalescent cattle. These data support the use of highly sensitive molecular assays into field settings for simple and rapid detection of FMDV.

## Introduction

Foot‐and‐mouth disease (FMD) is a highly infectious vesicular disease affecting both domesticated and wild cloven‐hooved animals. Caused by FMD virus (FMDV), FMD affects over 100 countries worldwide, with disease distribution roughly reflecting economic development (Jamal and Belsham, 2013). Although the case‐fatality rate of FMD is generally below 5%, the disease can be economically devastating: the annual global impact of FMD in terms of production losses and vaccination in endemic regions alone is estimated between US$ 6.5 and 21 billion (Knight‐Jones and Rushton, 2013). Furthermore, endemic infection represents a constant threat for FMD‐free countries, with outbreaks incurring severe economic losses: for example the UK 2001 outbreak is estimated to have cost the national economy US$ 9.2 billion (FAO, 2002). Early identification of FMDV in susceptible host populations is essential to minimize the impacts of FMD. Confirmation of FMD usually occurs at reference laboratories (OIE, 2012), although transport of specimens to these facilities can delay rapid real‐time decision making. The development of technologies to provide rapid, sensitive and *in situ* FMD diagnosis is therefore an ongoing research priority.

A number of developments have been made in regard to portable field assays for FMD diagnosis. Viral antigen detection is possible using portable immunochromatographic lateral flow devices (Ag‐LFDs), which have equivalent diagnostic sensitivity to the laboratory‐based antigen enzyme‐linked immunosorbent assay (ELISA) (Ferris et al., 2009, 2010). Although results are readable in as little as 10 min, Ag‐LFDs have only been validated for use with epithelial samples. Furthermore, low analytical sensitivity restricts their usefulness to the acute clinical phase of FMD, where epithelial samples contain high amounts of intact virus particles. The World Organisation for Animal Health (OIE) recommended real‐time reverse transcription polymerase chain reaction (rRT‐PCR) (Callahan et al., 2002) has been transferred onto a portable platform, the Enigma Field Laboratory (Enigma FL) (Enigma Diagnostics Limited, Salisbury, UK), which integrates silica paramagnetic bead‐based nucleic acid extraction, thermal cycling and result reporting with minimal user intervention. Using wet reagents, this platform showed high concordance to the laboratory‐based rRT‐PCR (Madi et al., 2012).

Similar advancements have been made with reverse transcription loop‐mediated isothermal amplification (RT‐LAMP): a rapid nucleic acid amplification technique that utilizes a strand‐displacing polymerase, multiple primers and autocycling under isothermal conditions. Simple amplification and detection methods have been demonstrated successfully for a previously published pan‐serotypic FMDV RT‐LAMP assay (Dukes et al., 2006), by combining water bath incubation with end‐point molecular LFD visualization (Waters et al., 2014), showing similar sensitivity to laboratory‐based rRT‐PCR. Furthermore, the development and commercial availability of portable fluorometers, such as the Genie^®^ II (OptiGene Ltd., Horsham, UK), allows for objective *in situ* real‐time RT‐LAMP (Craw and Balachandran, 2012), improving upon subjective visual detection measures such as turbidity and colour‐change dyes (Mori et al., 2001; Bearinger et al., 2011; Yamazaki et al., 2013). An additional benefit of RT‐LAMP is the ability to detect FMDV in samples without the requirement for nucleic acid extraction, allowing simple sample preparation methods, such as dilution in nuclease‐free water to be employed (Waters et al., 2014).

Currently published FMDV rRT‐PCR and RT‐LAMP assays, even those proposed for use in field settings, have been validated using ‘wet’ reagents, which contain temperature‐sensitive enzymes incompatible for field deployment or emergency stockpiling by countries normally free from disease. Methods are now available to lyophilize reagents, already tested in a number of LAMP (Boehme et al., 2007; Mair et al., 2013) and PCR assays (Siegmund et al., 2005; Aitichou et al., 2008; Helb et al., 2010; Takekawa et al., 2012), with benefits including improved stability, storage and transportability. This study describes the lyophilization, laboratory validation and field testing in endemic settings (Tanzania and Kenya) of a previously published FMDV‐specific rRT‐PCR (Callahan et al., 2002; Madi et al., 2012) and RT‐LAMP assay (Dukes et al., 2006; Waters et al., 2014). Field results were also compared against the existing Ag‐LFD field‐based diagnostic test (Ferris et al., 2009).

## Materials and Methods

Laboratory work was carried out at The Pirbright Institute (UK), unless stated otherwise.

### Viruses and clinical samples for laboratory evaluation

To determine the effect of lyophilizing rRT‐PCR reagents, a comparison between wet and lyophilized reagents was performed on a decimal dilution series of FMDV RNA in 0.1 *μ*g/*μ*l carrier RNA. Virus was obtained from clarified bovine thyroid cell lysate infected with FMDV (O/UAE/2/2003) and RNA extracted using the MagNA Pure LC Total Nucleic Acid Isolation Kit (Roche, Burgess Hill, UK) as manufacturer's instructions.

Laboratory analysis of the Enigma FL was performed using non‐extracted archival clinical epithelial suspensions from the field. Samples were obtained from the World Reference Laboratory for FMD (WRLFMD, The Pirbright Institute, UK) and consisted of the following: A (TAN/60/2012), SAT1 (TAN/25/2012; TAN/22/2012) and one un‐typed (serotype not determined) sample (TAN/54/2012) from which no virus could be isolated but was positive by rRT‐PCR. Archival field epithelial suspensions (also from WRLFMD) were also used to evaluate the performance of RT‐LAMP and RT‐LAMP combined with lateral flow detection (RT‐LAMP‐LFD) lyophilized reagents on clinical samples and consisted of: A (IRN/24/2012; TUR/7/2013; TUR/4/2013), SAT1 (TAN/50/2012), SAT2 (TAN/14/2012; BOT/15/2012) and Asia1 (TUR/2/2013). All above epithelial suspensions were prepared at 10% (w/v) in M25 phosphate buffer (35 mm Na_2_HPO_4_, 5.7 mm KH_2_PO_4_, pH 7.6).

Archival experimental bovine sera (*n* = 19) and oesophageal‐pharyngeal (OP) fluid samples (*n* = 21) from cattle infected with FMDV (isolate O/UKG/34/2001) were used to expand on the work of Waters et al. (2014) and create simple sample preparation protocols. In these transmission studies, calves were either challenged directly (via intradermolingual injection) or indirectly (via housing with a donor) with FMDV O/UKG/34/2001. The samples tested were collected daily from initial infection until 6 days post challenge. This study had been previously approved by The Pirbright Institute ethical review committee under the Animal Scientific Procedures Act (ASPA) 1986.

Archival epithelial suspensions, used for laboratory analysis in East Africa (Sokoine University of Agriculture, Morogoro, Tanzania), were from The Tanzania Veterinary Laboratory Agency (TVLA, Dar es Salaam, Tanzania) and were prepared in phosphate‐buffered saline (pH 7.2). Samples represented the following serotypes and regions: O (Musoma Rural; Tabora; Roya; Njombe; Mara; Kilimanjaro; Mtwara), A (Kagera), SAT1 (Dar es Salaam; Morogoro), SAT2 (Morogoro) and one un‐typed sample.

### Preparation of RNA standards for evaluation of lyophilized RT‐LAMP reagents

The limit of detection for the RT‐LAMP and RT‐LAMP‐LFD reagents was established using FMDV RNA standards. The FMDV 3D region was amplified using previously described primers 5ʹ‐GGA CAG GAC ATG CTC TCA G‐3ʹ and 5ʹ‐CAG GAA ACA GCT ATG ACT TTT TTT TTT TTT TTT TTT TTG‐3ʹ (Valdazo‐González et al., 2012) from FMDV isolate O/UKG/35/2001. The subsequent PCR product was purified using the Illustra GFX DNA/gel clean‐up kit (GE) and inserted into a pGEM^®^‐T vector (Promega, Southampton, UK). Synthetic viral RNA transcripts were produced by *in‐vitro* transcription (MEGAscript^®^, Ambion^®^, Thermo Fisher Scientific, Loughborough, UK) followed by DNase digestion using TurboDNase (Ambion^®^). Transcripts were purified using MEGAclear^™^ clean‐up kit (Life Technologies^™^, Thermo Fisher Scientific) prior to quantification at A^260^ using a NanoDrop ND‐1000 spectrophotometer (Thermo Fisher Scientific). Transcripts were diluted in nuclease‐free water to give a decimal dilution series of 10^6^ to 10^−1^ copies, which were tested in duplicate.

### Clinical samples for field evaluation

Field studies were carried out in Tanzania and Kenya, with Ankole‐cross and Zebu‐cross cattle, where serum, OP fluid and mouth/foot epithelium (where possible) samples were collected across different stages of infection (acute, convalescent and recovered). Samples were also collected from cattle in the affected herds which were clinically negative at the time of sampling. In total, samples from 66 individual cattle from 12 farms across East Africa were analysed *in situ*. This work comprised eight cattle from two Maasai small holdings from the Mvomero and Morogoro Rural Districts (Morogoro Region, Tanzania, June 2014), 41 cattle from seven small holdings located in the Serengeti District (Mara Region, Tanzania, October 2014) and 17 cattle from three farms in Nakuru County, Kenya (October 2013 and December 2014). Five of the cattle from Serengeti District, Tanzania were sampled on two separate occasions, 6 days apart. Locations were chosen following reports of FMDV infection. For Tanzania, field sampling was carried out with permission from the Tanzania Commission for Science and Technology (permit no. 2014‐368‐ER‐2005‐141) in accordance with ASPA guidelines. For Kenya, sampling was carried out as part of a training programme run by The European Commission for the Control of Foot‐and‐Mouth Disease (EuFMD). Samples were collected and processed as follows:

*OP fluid*: Was collected using a suitably sized probang cup following OIE guidelines (OIE, 2012). OP fluid was added neat to the mobile rRT‐PCR platform and diluted 1 in 10 in nuclease‐free water prior to analysis using RT‐LAMP and RT‐LAMP‐LFD (see below).
*Serum*: Cattle blood (10 ml) was collected from the jugular vein using Vacutainer^®^ Plus Plastic Serum Tubes (BD, Plymouth, UK). An aliquot was then centrifuged using an E8 field‐based centrifuge (LW Scientific) at 1400 ***g*** for 3 min at room temperature. Serum was added neat to the mobile rRT‐PCR platform and diluted 1 in 5 in nuclease‐free water prior to RT‐LAMP and RT‐LAMP‐LFD (see below).
*Epithelial tissue*: Epithelial tissue surrounding ruptured vesicles was collected from either the mouth or feet using sterile forceps and was prepared using the SVANODIP^®^ FMDV‐Ag Extraction Kit (Svanova, Boehringer Ingelheim, Uppsala, Sweden) according to the manufacturer's instructions. In brief, approximately 0.2 g of epithelial tissue was homogenized using the sample extraction vial in 1 ml of sample buffer from the SVANODIP^®^ FMDV‐Ag LFD kit (Svanova). The homogenate was left to settle for 1 min and the supernatant added neat to the mobile rRT‐PCR and Ag‐LFD platforms, and processed as previously described prior to RT‐LAMP by dilution 1 in 5 in nuclease‐free water (Waters et al., 2014) (see below).

### Real‐time reverse transcription PCR

#### Laboratory‐based OIE recommended rRT‐PCR

The diagnostic ‘gold‐standard’ one‐step rRT‐PCR was used to target the conserved 3D region of the FMDV genome, using primers and probes as previously described (Callahan et al., 2002). Reagents, parameters and thermal cycling were as reported in Shaw et al. (2007). All reactions were performed on nucleic acid extracted using the MagNA Pure LC Total Nucleic Acid Isolation Kit (Roche) and MagNA Pure LC automated platform as per manufacturer's guidelines (500 *μ*l : 200 *μ*l of sample and 300 *μ*l of lysis/binding buffer). Samples were assayed in duplicate on a bench top real‐time PCR machine (Stratagene Mx3005P^TM^; Agilent Technologies, Stockport, UK).

#### Enigma field laboratory

rRT‐PCR was performed as previously described (Madi et al., 2012), with primers and probes as published in Callahan et al. (2002). The platform integrates automated nucleic acid extraction (from 500 *μ*l sample), thermal cycling and result reporting. Within field settings, the Enigma FL was powered via a 15 V connection with vehicle auxiliary. Lyophilized reagents were supplied by Enigma Diagnostics Limited (Salisbury, Wiltshire), wet reagents were as above.

### Reverse transcription LAMP

#### Laboratory‐based

Reverse transcription LAMP was performed as previously described (Waters et al., 2014) with the following modifications. For wet reagents, the total reaction mixture of 25 *μ*l contained: 15 *μ*l isothermal master mix ISO‐001 (OptiGene Ltd.) primers and concentrations as per Dukes et al. (2006), 2 U AMV reverse transcriptase (New England Biolabs, Hitchin, UK), 5 *μ*l template and made up to total volume with nuclease‐free water. Lyophilized reagents were developed by OptiGene Limited, using isothermal master mix ISO‐001 with the addition of primers (as above), stabilizing sugars and AMV. Lyophilized pellets were re‐suspended with 15 *μ*l of re‐suspension buffer, 5 *μ*l sample and made up to 25 *μ*l total volume with nuclease‐free water. RT‐LAMP reactions were run at 65°C for the manufacturer's recommended 30 min on a Stratagene Mx3005P^™^, followed by assay inactivation at 85°C for 5 min. All samples were tested in duplicate.

#### Fluorescence detection

ISO‐001 contains an intercalating dye, enabling results to be visualized using fluorescence collected at 1 min intervals. A positive RT‐LAMP reaction was indicated by an exponential increase in fluorescence (δR) and the time to positivity (*T*_P_) was defined when reactions reached a threshold increase of δR 1000. To confirm that amplicons were FMDV‐specific, annealing analysis was performed on RT‐LAMP products using the Genie^®^ II (OptiGene Ltd.). LAMP products were heated to 98°C, then cooled to 80˚C ramping at 0.05°C/s. Anneal temperature (*T*_a_) calculations were automated using Genie^®^ Explorer v0.2.1.1 software (OptiGene Ltd.). Samples were called positive if amplification had occurred and the LAMP product annealed in the FMDV amplicon‐specific temperature range 87.5–89.5°C (88.5°C was the average *T*_a_ over 210 FMDV‐positive RT‐LAMP reactions, with 98.55% of reactions within ±1°C).

#### RT‐LAMP combined with lateral flow detection

The RT‐LAMP‐LFD assay was modified as previously described by labelling the 5′ termini of the inner LAMP primers (FIP and BIP) (Waters et al., 2014). Results were visualized using PCRD‐2 lateral flow devices (Abingdon Health, York, UK) as per manufacturer's instructions. A positive result was signified by the presence of two blue bands (test and LFD control line); negative results were indicated by a single band (the LFD control line). For all images shown, the upper band represents the LFD control line and lower band the test line with respect to the loading pad at the bottom.

#### Field‐based RT‐LAMP and RT‐LAMP‐LFD

Mobile RT‐LAMP and RT‐LAMP‐LFD were performed on the Genie^®^ II using the lyophilized reagents as described above. *T*_P_ and *T*_a_ calculations were automated using Genie^®^ Explorer v0.2.1.1 software.

### Antigen LFD

Six drops of homogenized epithelium from the SVANODIP^®^ FMDV‐Ag Extraction Kit were added to the SVANODIP^®^ FMDV‐Ag LFDs as previously published (Ferris et al., 2009) and following manufacturer's guidelines. LFDs were incubated for 10 min at ambient temperature prior to interpretation of results.

### Statistical analysis

Cohen's Kappa statistic (κ) and the proportion of observed agreement (*A*_obs_) were used to measure the agreement between diagnostic tests. All statistical tests were performed in the statistical package R (R Core Team, 2014). Cohen's Kappa statistic (κ) was interpreted as published in Landis and Koch (1977).

## Results

### Laboratory evaluation of lyophilized rRT‐PCR reagents

The wet Enigma reagents had been previously reported to equal the limit of detection (between 10 and 100 viral genome copies) to the laboratory‐based rRT‐PCR (Madi et al., 2012). Lyophilization of reagents did not adversely affect the performance of the assay, with both lyophilized and wet reagents detecting down to 10^−6^ of the dilution series (data not shown).

Archival epithelial suspensions were then used to evaluate the full Enigma FL protocol (integrated nucleic acid extraction and rRT‐PCR) incorporating lyophilized reagents for RNA extraction and rRT‐PCR. Results reported from the Enigma FL were comparable to results gained using the OIE recommended rRT‐PCR performed on MagNA Pure extracted nucleic acid (Table 1).

**Table 1:** Evaluation of lyophilized Enigma FL reagents using clinical samples. Comparison between cycle threshold (*C*_T_) values for (a) rRT‐PCR performed on extracted RNA from epithelial suspensions using wet reagents on a bench top real‐time PCR machine; (b) rRT‐PCR performed on neat epithelial suspensions using lyophilized rRT‐PCR reagents on the Enigma FL

Serotype	A	SAT1		SND	Negative
Sample ID	TAN/60/2012	TAN/25/2012	TAN/22/2012	TAN/54/2012	Epithelium
(a) rRT‐PCR (*C*_T_)	38.83	14.45	14.30	26.27	–
(b) Enigma Report	Positive	Positive	Positive	Positive	Negative

SND, Serotype not determined.

### Laboratory evaluation of lyophilized RT‐LAMP and RT‐LAMP‐LFD reagents

**Figure 1:**
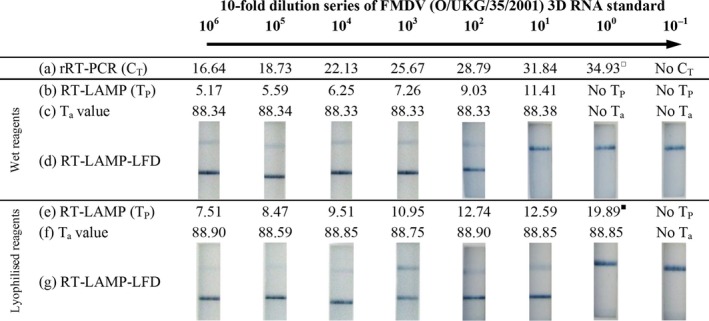
Limit of detection analysis for RT‐LAMP and RT‐LAMP‐LFD. Data show (a) rRT‐PCR *C*_T_ values; (b) wet RT‐LAMP *T*_P_ values; (c) anneal analysis of (b); (d) wet RT‐LAMP‐LFD results; (e) lyophilized RT‐LAMP *T*_P_ values; (f) anneal analysis of (e); (g) lyophilized RT‐LAMP‐LFD results. For rRT‐PCR ^□^ represents *C*_T_ values greater than the diagnostic threshold of C_T_ <32 as reported by Shaw et al. (2007); for RT‐LAMP ^■^ indicates that out of the identical duplicates, one was positive and the other negative. For RT‐LAMP‐LFD, a positive result is indicated by the presence of two lines (lower test line and upper control line), whereas a negative result only generates a single band (upper control line) with respect to the loading pad at the bottom (not shown).

The FMDV RT‐LAMP wet assay has been previously reported to have an analytical sensitivity of 10^1^ copies/*μ*l (Dukes et al., 2006). Using RNA standards, equivalent results were evident for lyophilized RT‐LAMP and RT‐LAMP‐LFD, consistently detecting down to 10^1^ copies/*μ*l (Fig. 1). A *T*_P_ value is not given for RT‐LAMP‐LFD due to the interference of the fluorescein‐labelled inner primer (required for detection with LFDs) with the intercalating dye used for RT‐LAMP detection on the Genie^®^ II.

To evaluate the performance of RT‐LAMP/RT‐LAMP‐LFD lyophilized reagents on clinical samples, the previously published protocol was used to prepare epithelial suspensions (Waters et al., 2014) prior to direct use in RT‐LAMP/RT‐LAMP‐LFD. Samples represented five FMDV serotypes (O, A, SAT1, SAT2 and Asia 1) and positive amplification was observed in both assays in all five cases (data not shown).

### Determination of simple protocols for preparation of clinical samples prior to RT‐LAMP

Archival serum and OP fluid samples were used to expand on the work of Waters et al. (2014) to create simple sample preparation protocols. Samples were added to RT‐LAMP (wet reagents) following MagNA Pure nucleic acid extraction or dilution in nuclease‐free water (Table 2). As a reference, nucleic acid extracted from all samples was assayed using the OIE recommended rRT‐PCR (Callahan et al., 2002). High agreement was apparent between rRT‐PCR and RT‐LAMP test results for sera following nucleic acid extraction (κ = 1.000, *P* = 0.000, *A*_obs_ = 1.000) and 1 in 5 dilution (κ = 0.791, *P* = 0.000, *A*_obs_ = 0.895). Adding sera neat to RT‐LAMP resulted in an inhibitory effect with no amplification seen in any of the rRT‐PCR positive samples. Diluting sera one in five gave the optimum results for RT‐LAMP, therefore this dilution was used for subsequent sera samples. Similar results were seen when comparing rRT‐PCR and RT‐LAMP results for OP fluid following MagNA Pure nucleic acid extraction (κ = 0.859, *P* = 0.002, *A*_obs_ = 0.952) and 1 in 5 dilutions (κ = 0.577, *P* = 0.029, *A*_obs_ = 0.857). When OP fluid was added to RT‐LAMP neat, 16/16 RT‐LAMP negatives showed non‐specific amplification, which was still evident in 1 in 5 dilutions (as determined by incorrect anneal temperatures). OP fluid samples were therefore diluted 1 in 10 and compared to rRT‐PCR results (κ = 0.741, *P* = 0.005, *A*_obs_ = 0.905). At this dilution, results were optimal and no non‐specific amplification was evident. Subsequent OP fluid samples were diluted 1 in 10 in nuclease‐free water.

**Table 2:** Comparative tables between the OIE recommended rRT‐PCR and RT‐LAMP. The following sample preparations were trialled for RT‐LAMP (wet reagents): (a) extracted RNA from sera; (b) neat sera; (c) 1 in 5 dilutions of sera; (d) extracted RNA from OP fluid; (e) neat OP fluid; (f) 1 in 5 dilutions of OP fluid; (g) 1 in 10 dilutions of OP fluid. Data in tables represents the numbers of samples tested. Cohen's Kappa statistic (κ), *P*‐value and the proportion of observed agreement (*A_obs_*) are reported

	(a) Sera extracted RNA			(b) Neat sera			(c) Sera 1 in 5		
	RT‐LAMP +	RT‐LAMP−	Total	RT‐LAMP +	RT‐LAMP−	Total	RT‐LAMP +	RT‐LAMP−	Total
rRT‐PCR +	11	0	11	0	11	11	9	2	11
rRT‐PCR−	0	8	8	0	8	8	0	8	8
Total	11	8	19	0	19	19	9	10	19
	κ = N/A, *P* = N/A, *A*_obs_ = 1.000			κ = 0.000, *P* = 0.500, *A*_obs_ = 0.421			κ = 0.79, *P* = 0.000, *A*_obs_ = 0.895		

	(d) OP fluid extracted RNA			(e) Neat OP fluid			(f) OP fluid 1 in 5			(g) OP fluid 1 in 10		
	RT‐LAMP +	RT‐LAMP−	Total	RT‐LAMP+	RT‐LAMP−	Total	RT‐LAMP+	RT‐LAMP−	Total	RT‐LAMP+	RT‐LAMP−	Total
rRT‐PCR +	16	1	17	5	12^a^	17	15	2	17	15	2	17
rRT‐PCR−	0	4	4	0	4^a^	4	1	3^a^	4	0	4	4
Total	16	5	21	5	16	21	16	5	21	15	6	21
	κ = 0.859, *P* = 0.002, *A*_obs_ = 0.952			κ = 0.137, *P* = 0.190, *A*_obs_ = 0.429			κ = 0.577, *P* = 0.029, *A*_obs_ = 0.857			κ = 0.741, *P* = 0.005, *A*_obs_ = 0.905		

a Groups in which at least one reaction showed non‐specific amplification in RT‐LAMP. rRT‐PCR was performed on nucleic acid extracted using the MagNA Pure and a diagnostic threshold of *C*_T_ < 32 (Shaw et al., 2007) was used to distinguish between rRT‐PCR positive and negative samples.

### Detection of FMDV in endemic laboratory settings using lyophilized rRT‐PCR, RT‐LAMP and RT‐LAMP‐LFD assays

**Figure 2:**
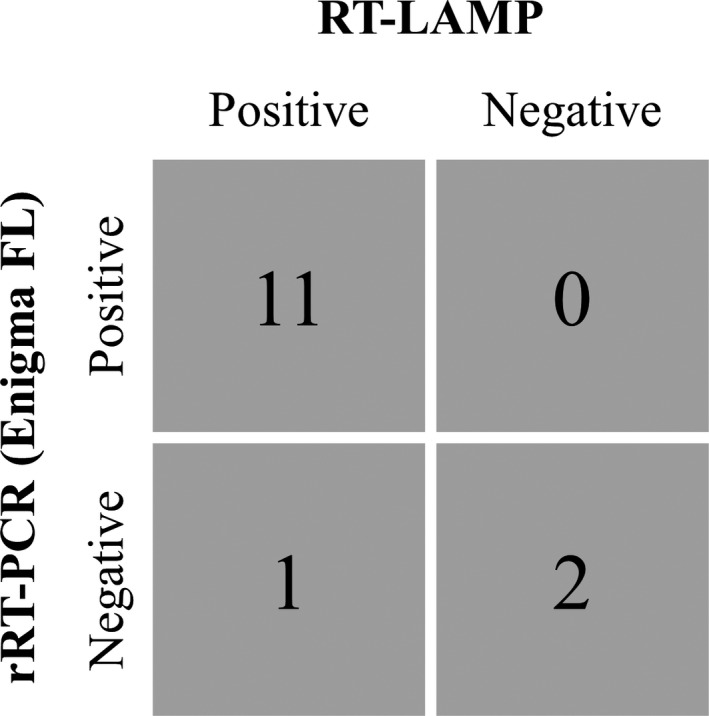
Mobile detection of FMDV by rRT‐PCR and RT‐LAMP (100% agreement was evident between RT‐LAMP‐LFD and fluorescence‐based RT‐LAMP for these samples using the Genie^®^ II). Detection was performed using 14 epithelial samples submitted to The Tanzanian Veterinary Laboratory Agency (TVLA). Tests were performed in a local laboratory at Sokoine University of Agriculture (SUA) in Morogoro, Tanzania.

Fourteen archival epithelial suspensions, representing four FMDV serotypes and 10 locations across Tanzania, were used to compare the performance of portable diagnostic systems on clinical samples (Genie^®^ II for RT‐LAMP/RT‐LAMP‐LFD, Enigma FL for rRT‐PCR and Ag‐LFDs) within a local laboratory setting in a FMD endemic region. 100% agreement was evident between RT‐LAMP and RT‐LAMP‐LFD assay results, which were both in high agreement with rRT‐PCR results (κ = 0.759, *P* = 0.033, *A*_obs_ = 0.929) (Fig. 2). Ag‐LFDs showed reduced sensitivity with 3/12 samples called positive by RT‐LAMP/RT‐LAMP‐LFD reported as negative by Ag‐LFD.

### Detection of FMDV *in situ* using lyophilized rRT‐PCR in Kenya

Following successful evaluation of the Enigma FL on archived field samples within a laboratory setting, preliminary field testing was performed in Nakuru, Kenya (October 2013) on 10 field samples (four blood; five epithelium; one vesicular fluid) from six cattle, in two locations. In all cases, mobile rRT‐PCR results were consistent with clinical observations (Table 3).

**Table 3:** Initial Enigma FL rRT‐PCR field testing results. Samples comprised blood, epithelium and vesicular fluid, collected from six cattle located in Nakuru, Kenya. Ag‐LFD results, performed on epithelial suspensions, are shown for comparison

Animal number	Location 1				Location 2	
	1	2	3	4	5	6
Temperature (°C)	38.8	39.6	36.5	Not tested	40.5	39.5
Lesion age	6–7 days	2–3 days	None present	3 days	1–2 days	2–3 days
Samples collected	Blood	Blood	Blood	Epithelium	Blood	Epithelium
	Epithelium	Epithelium			Epithelium	
					Vesicular fluid	
Enigma FL (*C*_T_)	Blood: *C*_T_ 26	Blood: *C*_T_ 18	Blood: *C*_T_ 32	Epithelium: *C*_T_ 30	Blood: *C*_T_ 18	Epithelium: *C*_T_ 16
	Epithelium: *C*_T_ –ve	Epithelium: *C*_T_ 31			Epithelium: *C*_T_ 32	
					Vesicular fluid: *C*_T_ 16	
Ag‐LFD	Negative	Positive	Not applicable	Negative	Positive	Positive

### Detection of FMDV *in situ* using lyophilized RT‐LAMP, RT‐LAMP‐LFD and rRT‐PCR assays

**Figure 3:**
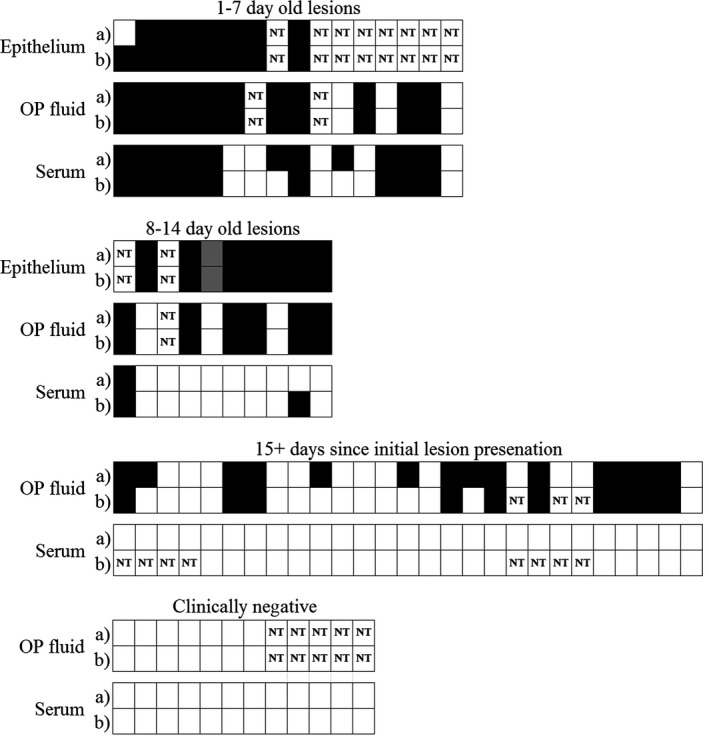
*In situ* (a) RT‐LAMP and (b) RT‐LAMP‐LFD results for 144 East African samples. Cattle were either acutely infected with FMD, displayed healing FMD lesions, were clinically recovered from FMD or were FMD negative. Black: positive result; white: negative result; ‘NT’: reaction not performed. Each column represents one animal; rows represent sample type. For some animals more than one epithelial sample was tested, grey squares represent a mix of positive and negative results.

The Genie^®^ II and RT‐LAMP/RT‐LAMP‐LFD protocols devised in the laboratory were tested on 144 samples from 60 cattle (multiple samples taken from each animal, representing epithelium, serum and OP fluid) across 10 farms in East Africa (five cattle sampled on two occasions) and compared to FMD clinical presentation. Of the cattle that displayed approximately1–7 day old lesions, RT‐LAMP identified the presence of FMDV in 13 epithelial, 11 OP fluid and 11 sera samples, the remaining samples were negative, consistent with clinical observations (e.g. clearance of viraemia). Of the cattle approximately 8–14 days post initial lesion presentation, RT‐LAMP identified FMDV in eight epithelial, six OP fluid samples and one serum sample; the remaining samples were negative, consistent with disease progression. Of the clinically recovered cattle (approximately 15+ days post initial lesion presentation), RT‐LAMP identified FMDV in 14 OP fluid samples (consistent with delayed FMDV clearance), while all serum samples were negative. Of the 12 clinically negative cattle sampled, all OP fluid and sera samples were negative (Fig. 3). High agreement was evident between RT‐LAMP and RT‐LAMP‐LFD for all sample types: sera (κ = 0.837, *P* = 0.000, *A*_obs_ = 0.947), OP fluid (κ = 0.852, *P* = 0.000, *A*_obs_ = 0.926) and epithelial samples (κ = 0.646, *P* = 0.123, *A*_obs_ = 0.957). All test results were consistent with clinical observations.

**Figure 4:**
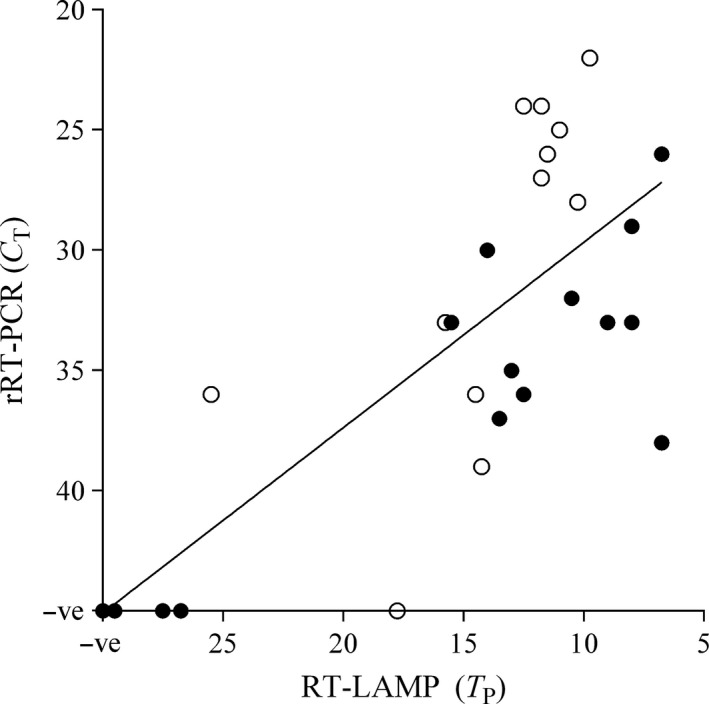
Comparison between rRT‐PCR (Enigma FL) and RT‐LAMP (Genie^®^ II) on field samples tested *in situ* within Tanzania (Serengeti District and Morogoro). (○) OP fluid samples; (●) epithelial samples. The linear regression (*R* ^2^ = 0.5207) between rRT‐PCR *C*_T_ and fluorescence‐based RT‐LAMP *T*_P_ is displayed.

For comparison, 34 of the samples assayed on RT‐LAMP and RT‐LAMP‐LFD above (13 epithelium; 17 OP fluid; 4 sera) were also assayed on rRT‐PCR with the Enigma FL in the field. Fair agreement was present between molecular platforms: RT‐LAMP and rRT‐PCR (κ = 0.635, *P* = 0.001, *A*_obs_ = 0.853) and RT‐LAMP‐LFD and rRT‐PCR (κ = 0.781, *P* = 0.000, *A*_obs_ = 0.912) (Fig. 4). In addition, 23 epithelial samples were also assayed using Ag‐LFDs, with only slight agreement evident between both RT‐LAMP and Ag‐LFD (κ = 0.008, *P* = 0.486, *A*_obs_ = 0.522) and RT‐LAMP‐LFD and Ag‐LFD results (κ = 0.095, *P* = 0.332, *A*_obs_ = 0.565). Of 13 epithelial samples assayed by both rRT‐PCR and Ag‐LFD, 8/13 results showed agreement, with 5/12 rRT‐PCR positive samples negative by Ag‐LFD (Appendix S1).

Four clinical samples (two epithelial and two vesicular fluid) from two of the acutely infected cattle tested (tag numbers 7804 and 7805) in the Serengeti District, Tanzania were also shipped to WRLFMD for confirmation of FMD. All four samples were confirmed positive for FMD using rRT‐PCR and were typed as serotype SAT1 by antigen ELISAs (data not shown).

## Discussion

Robust rapid diagnosis of FMD is essential for the effective implementation of disease monitoring, control and eradication strategies, particularly during incursions into disease free countries (Anderson, 2002). Historically where such diagnostics have not been available, precautionary control strategies including ‘slaughter on suspicion’ have been implemented to bypass lengthy laboratory confirmation and limit the potential size of the outbreak. However in the case of the UK 2001 FMD outbreak, this approach led to unnecessary culling, with retrospective analysis showing no evidence of FMDV on 23% of premises designated as infected (Ferris et al., 2001). This manuscript describes the evaluation of lyophilized rRT‐PCR and RT‐LAMP assays to rapidly detect FMDV directly from clinical samples in laboratory and field settings.

Lyophilization of reagents had no impact on the performance of either assay, with fluorescence‐based RT‐LAMP, RT‐LAMP‐LFD and rRT‐PCR maintaining comparable analytical sensitivity to the equivalent ‘wet’ reagents and laboratory‐based rRT‐PCR. However, a one log_10_ reduction in the analytical sensitivity of wet RT‐LAMP‐LFD reagents was evident comparatively to fluorescence‐based RT‐LAMP. This was observed at the threshold of analytical sensitivity and is therefore likely due to the quantity of amplicon‐latex bead complexes being too low for visualization. Analytical sensitivity for this assay could be increased if required (e.g. for samples with a low viral titer) by extending the incubation period from the 30 min used in this study to the 60 min previously reported by Waters et al. (2014). Furthermore, simple sample preparation methods for RT‐LAMP and RT‐LAMP‐LFD (Waters et al., 2014) were expanded further to include sera and OP fluid, improving the diagnostic potential of the assays. This study focuses on epithelium, OP fluid and serum samples, however, other sample types such as milk and swabs may also be suitable for FMD diagnosis.

When deployed for field validation, both RT‐LAMP and rRT‐PCR assays generated results consistent with clinical observations, enabling virus to be detected across the FMD clinical window from acute infection to delayed viral clearance. In total, samples from 66 cattle across 12 endemic field settings within East Africa were tested. The early detection of FMDV was further substantiated by the data from experimental samples, where positive results were generated as early as 1 day post challenge. In all these studies, molecular assays consistently outperformed Ag‐LFDs by their ability to detect virus at lower concentrations and in a greater number of clinical samples. However, Ag‐LFDs remain useful for confirmation of FMD positive animals during the acute stage of clinical infection (using epithelial samples) and were consistent with molecular assay results under these circumstances.

Field validation highlighted a number of important factors to consider for future protocol design specific to the use of RT‐LAMP and RT‐LAMP‐LFD *in situ*. For example appropriate sample collection is required to ensure (i) sufficient amount of material is available for processing and (ii) samples collected are not contaminated with soil (epithelium) or blood/bolus (OP fluid). Although LAMP is consistently reported to show increased tolerance to inhibitors comparatively to PCR (Poon et al., 2006; Waters et al., 2014), high levels of contaminants in samples are likely to increase false‐negative (reaction inhibition) or false‐positive results (non‐specific amplification). This is of particular concern when considering the ability of tests to confirm FMD negative animals and the use of OP fluid samples used to detect carrier status. Therefore, work is required to further improve sample preparation methods for incorporation into field protocols.

During field validation, an initial period of time was spent in local laboratories to confirm that reagents and equipment were suitable for use post‐air travel. For this, 14 archival epithelial suspensions were utilized. This process was undertaken with local laboratory staff and highlighted the additional potential of these technologies to improve local diagnostic capacity within endemic settings. At present, laboratories within these settings are often confounded by limited laboratory capacity (skilled personnel and availability of technologies/consumables) and poor transport links (maintenance of the cold chain). The provision of lyophilized reagents within disposable consumables, in addition to simple reporting procedures, helps to address these issues by (i) negating the need to order reagents and consumables from multiple suppliers, (ii) simplifying reagent storage requirements and (iii) minimizing user intervention, thus opening up sensitive molecular technologies to unskilled staff. Combined, this would enable countries to progress along the FAO progressive control pathway for FMD eradication (Namatovu et al., 2013).

In conclusion, we present the development and evaluation of lyophilized FMDV‐specific rRT‐PCR and RT‐LAMP assays, which both maintained similar analytical sensitivity to the OIE recommended rRT‐PCR. Both platforms were highly compatible with field use, the Enigma FL (rRT‐PCR) through integration of RNA extraction, and RT‐LAMP through robust chemistry conditions negating the requirement for RNA extraction. Therefore, this study demonstrates an important transition for FMDV‐specific molecular assays into formats suitable for field diagnostic use.

## Supporting information


**Appendix S1.** Results for preliminary field trials.
