# Supplementary material for: Evaluation of Two Lyophilized Molecular Assays to Rapidly Detect Foot‐and‐Mouth Disease Virus Directly from Clinical Samples in Field Settings
Source: Transbound Emerg Dis. 2015 Nov 30;64(3):861–71. doi: 10.1111/tbed.12451 (PMC5434942; doi:10.1111/tbed.12451)
Supplement: Supplementary file 1 — Appendix S1. Results for preliminary field trials. [file TBED-64-861-s001.docx]

Appendix **1**. Results for preliminary field trials

| Animal reference | Location | Time since start of clinical signs | Clinical signs | Heart rate (bpm) | (°C) Rectal temperature | Sample | Antigen LFD | Enigma FL | RT-LAMP Tp | RT-LAMP T_a_ | RT-LAMP-LFD | RT-LAMP-LFD T_a_ |
| --- | --- | --- | --- | --- | --- | --- | --- | --- | --- | --- | --- | --- |
| Mcow1 | Morogoro, Tanzania | ca. one month | none | N/A | N/A | serum | N/A | - | - | - | N/A | - |
|  |  |  |  |  |  | OP fluid | + | 33 | 15.50 | 88.81 | + | 88.16 |
| Mcow2 | Morogoro, Tanzania | ca. one month | none | N/A | N/A | serum | N/A | - | - | - | N/A | - |
|  |  |  |  |  |  | OP fluid | - | - | 29.5 | 88.5 | - | 88.29 |
| Mcow3 | Morogoro, Tanzania | ca. one month | none | N/A | N/A | serum | N/A | - | - | - | N/A | - |
|  |  |  |  |  |  | OP fluid | N/A | - | - | - | - | - |
| Mcow4 | Morogoro, Tanzania | ca. one month | none | N/A | N/A | serum | N/A | - | - | - | N/A | - |
|  |  |  |  |  |  | OP fluid | N/A | - | - | - | - | - |
| Mcow5 | Morogoro, Tanzania | ca. two months | none | N/A | N/A | serum | N/A | N/A | - | - | N/A | - |
|  |  |  |  |  |  | OP fluid | N/A | N/A | - | - | N/A | - |
| Mcow6 | Morogoro, Tanzania | ca. two months | bleeding gums | N/A | N/A | serum | N/A | N/A | - | - | N/A | - |
|  |  |  |  |  |  | OP fluid | + | 38 | 6.75 | 88.75 | + | 88.71 |
| Mcow7 | Morogoro, Tanzania | ca. two months | none | N/A | N/A | serum | N/A | N/A | - | - | N/A | - |
|  |  |  |  |  |  | OP fluid | N/A | N/A | - | - | N/A | - |
| Mcow8 | Morogoro, Tanzania | ca. two months | none | N/A | N/A | serum | N/A | N/A | - | - | N/A | - |
|  |  |  |  |  |  | OP fluid | N/A | N/A | - | - | N/A | - |
| 7647 | Serengeti, Tanzania | ca. 7 days | lesions, lameness and salivation | 21 | 39.9 | serum | N/A | N/A | - | - | - | - |
|  |  |  |  |  |  | OP fluid | N/A | N/A | 30.00 | - | - | - |
| 7801 | Serengeti, Tanzania | ca. 4 days | severe foot and mouth lesions salivation and lameness | 26 | 36.8 | serum | N/A | N/A | 20.75 | 88.90 | + | 89.34 |
|  |  |  |  |  |  | OP fluid | - | N/A | 26.25 | 88.49 | + | 89.34 |
|  |  |  |  |  |  | LF epi | + | 28 | 10.25 | 88.93 | + | 88.93 |
|  |  |  |  |  |  | LH epi | + | N/A | 9.75 | 88.98 | + | 89.38 |
|  |  |  |  |  |  | RF epi | + | N/A | 9.75 | 89.04 | + | 89.63 |
|  |  |  |  |  |  | RH epi | + | N/A | 10.50 | 89.09 | + | 89.58 |
|  |  |  |  |  |  | gum epi | - | N/A | 11.75 | 88.94 | + | 89.53 |
| 7802 | Serengeti, Tanzania | ca. 1 days | severe foot and mouth lesions salivation and lameness | 23 | 38.1 | serum | N/A | N/A | 23.00 | 88.84 | + | 89.05 |
|  |  |  |  |  |  | OP fluid | - | N/A | 11.25 | 89.04 | + | 88.84 |
|  |  |  |  |  |  | RF epi | - | N/A | 13.75 | 89.43 | + | 89.59 |
|  |  |  |  |  |  | gum epi | - | - | 17.75 | 88.96 | + | 88.94 |
|  |  | ca. 7 days | healing lesions | 30 | 36.9 | serum | N/A | N/A | 22.50 | 88.27 | + | 88.77 |
|  |  |  |  |  |  | OP fluid | - | 37 | 13.5 | 88.95 | + | 89.13 |
| 7803 | Serengeti, Tanzania | ca. 1 days | severe foot lesions, salivation and lameness | 37 | 38.5 | serum | N/A | N/A | 17.75 | 89.40 | + | 89.41 |
|  |  |  |  |  |  | OP fluid | - | N/A | 13.00 | 89.44 | + | 89.30 |
|  |  |  |  |  |  | LF epi | + | 27 | 11.75 | 89.44 | + | 88.99 |
|  |  |  |  |  |  | LH vf | N/A | N/A | 18.00 | 89.43 | + | 89.34 |
|  |  | ca. 7 days | healing lesions | 31 | 37.5 | serum | N/A | N/A | 25.00 | 88.37 | + | 89.07 |
|  |  |  |  |  |  | OP fluid | - | 35 | 13.00 | 89.05 | + | 89.07 |
| 7804 | Serengeti, Tanzania | ca. 2 days | severe foot and mouth lesions salivation and lameness | 36 | 38.4 | serum | N/A | N/A | 13.25 | 89.39 | + | 89.39 |
|  |  |  |  |  |  | OP fluid | + | N/A | 11.25 | 89.35 | + | 89.10 |
|  |  |  |  |  |  | RH epi | + | 22 | 9.75 | 88.98 | + | 88.89 |
|  |  |  |  |  |  | RF vf | N/A | N/A | 23.50 | 89.44 | + | 88.79 |
|  |  | ca. 8 days | healing lesions | 28 | 37.1 | serum | N/A | N/A | - | - | - | - |
|  |  |  |  |  |  | OP fluid | N/A | N/A | - | - | - | - |
|  |  |  |  |  |  | gum epi | - | 33 | 15.75 | 88.67 | + | 88.67 |
| 7805 | Serengeti, Tanzania | ca. 1 day | severe foot lesions, salivation and lameness | 38 | 39.9 | serum | N/A | N/A | 17.50 | 88.93 | + | 88.88 |
|  |  |  |  |  |  | OP fluid | - | N/A | 17.50 | 89.24 | + | 89.29 |
|  |  |  |  |  |  | LH epi | + | 26 | 11.50 | 89.68 | + | 88.98 |
|  |  |  |  |  |  | LF vf | N/A | N/A | 25.00 | 89.24 | + | 89.49 |
|  |  | ca. 7 days | healing lesions | 37 | 37.0 | serum | N/A | N/A | - | - | - | - |
|  |  |  |  |  |  | OP fluid | - | 36 | 12.50 | 89.16 | + | 89.10 |
| 7806 | Serengeti, Tanzania | ca. 1 days | severe foot lesions, salivation and lameness | 24 | 38.3 | serum | N/A | N/A | 25.00 | 88.90 | + | 89.25 |
|  |  |  |  |  |  | OP fluid | + | N/A | 7.50 | 89.39 | + | 89.29 |
|  |  |  |  |  |  | LH epi | + | N/A | 12.75 | 89.25 | + | 89.54 |
|  |  |  |  |  |  | gum epi | + | N/A | 11.00 | 88.88 | + | 89.33 |
|  |  | ca. 7 days | healing lesions | 26 | 37.6 | serum | N/A | N/A | 25.00 | 88.35 | + | 88.95 |
|  |  |  |  |  |  | OP fluid | N/A | N/A | - | - | - | - |
| 7818 | Serengeti, Tanzania | N\A | N\A | 25 | 35.5 | serum | N/A | N/A | - | - | - | - |
|  |  |  |  |  |  | OP fluid | N/A | N/A | - | - | - | - |
| 7807 | Serengeti, Tanzania | ca. 2 weeks | healing lesions and lameness | 23 | 36.9 | serum | N/A | N/A | - | - | - | - |
|  |  |  |  |  |  | OP fluid | - | N/A | 13.25 | 88.95 | + | 88.91 |
|  |  |  |  |  |  | gum epi | - | 36 | 14.50 | 89.15 | + | 89.10 |
| 7808 | Serengeti, Tanzania | ca. 2 weeks | healing lesions and lameness | 21 | 36.8 | serum | N/A | N/A | - | - | - | - |
|  |  |  |  |  |  | OP fluid | N/A | N/A | - | - | - | - |
|  |  |  |  |  |  | RF epi | + | 24 | 11.75 | 88.89 | + | 88.69 |
|  |  |  |  |  |  | gum epi | - | N/A | - | - | - | - |
| 7809 | Serengeti, Tanzania | ca. 2 weeks | healing lesions, lameness and salivation | 29 | 36.4 | serum | N/A | N/A | - | - | - | - |
|  |  |  |  |  |  | OP fluid | + | N/A | 17.75 | 88.85 | + | 88.82 |
|  |  |  |  |  |  | tongue epi | + | 25 | 11.00 | 89.23 | + | 88.99 |
| 7810 | Serengeti, Tanzania | ca. 2 weeks | healing lesions and lameness | 23 | 37.4 | serum | N/A | N/A | - | - | - | - |
|  |  |  |  |  |  | OP fluid | - | N/A | 8.50 | 89.3 | + | 89.02 |
|  |  |  |  |  |  | gum epi | - | 24 | 12.50 | 88.95 | + | 88.74 |
| 7811 | Serengeti, Tanzania | ca. 2 weeks | healing lesions | 23 | 37.6 | serum | N/A | N/A | 19.25 | 88.78 | + | 88.84 |
|  |  |  |  |  |  | OP fluid | - | N/A | 8.75 | 89.29 | + | 89.29 |
| 7812 | Serengeti, Tanzania | ca. 2 weeks | healing lesions and salivation | 24 | 38.3 | serum | N/A | N/A | - | - | - | - |
|  |  |  |  |  |  | OP fluid | N/A | N/A | - | - | - | - |
|  |  |  |  |  |  | gum epi | - | 36 | 25.50 | 89.44 | + | 89.25 |
| 7813 | Serengeti, Tanzania | ca. 2 weeks | healing lesions and lameness | 26 | 37.6 | serum | N/A | N/A | - | - | + | 88.87 |
|  |  |  |  |  |  | OP fluid | - | N/A | 13.50 | 89.25 | + | 88.90 |
|  |  |  |  |  |  | gum epi | - | 39 | 14.25 | 88.98 | + | 88.84 |
| 7814 | Serengeti, Tanzania | ca. 2 weeks | healing lesions and lameness | 28 | 39.4 | serum | N/A | N/A | - | - | - | - |
|  |  |  |  |  |  | OP fluid | + | N/A | 13.50 | 88.90 | + | 89.15 |
|  |  |  |  |  |  | RH epi | + | 24 | 12.50 | 89.38 | + | 88.84 |
| 7730 | Serengeti, Tanzania | ca. 2 months | healed mouth and foot lesions | 25 | 38.0 | serum | N/A | N/A | - | - | - | - |
|  |  |  |  |  |  | OP fluid | - | 29 | 8.00 | 88.83 | + | 88.92 |
| 7731 | Serengeti, Tanzania | ca. 2 months | healed mouth lesion | 22 | 37.9 | serum | N/A | N/A | - | - | - | - |
|  |  |  |  |  |  | OP fluid | + | 26 | 6.75 | 88.93 | + | 88.92 |
| 7743 | Serengeti, Tanzania | ca. 2 months | none | 22 | 38.5 | serum | N/A | N/A | - | - | - | - |
|  |  |  |  |  |  | OP fluid | + | 33 | 8.00 | 89.02 | + | 89.04 |
| 7744 | Serengeti, Tanzania | ca. 2 months | slight loss of condition | 23 | 38.3 | serum | N/A | N/A | - | - | - | - |
|  |  |  |  |  |  | OP fluid | - | 33 | 9.00 | 88.89 | + | 88.89 |
| 7746 | Serengeti, Tanzania | ca. 2 months | none | 21 | 37.6 | serum | N/A | N/A | - | - | - | - |
|  |  |  |  |  |  | OP fluid | N/A | - | 26.75 | 66.47 | - | 66.51 |
| 7732 | Serengeti, Tanzania | ca. 1 month | none | 19 | 38.5 | serum | N/A | N/A | - | - | - | - |
|  |  |  |  |  |  | OP fluid | N/A | N/A | - | - | - | - |
| 7733 | Serengeti, Tanzania | ca. 1 month | none | 20 | 38.2 | serum | N/A | N/A | - | - | - | - |
|  |  |  |  |  |  | OP fluid | - | - | 25.00 | 88.59 | + | 88.83 |
| 7734 | Serengeti, Tanzania | ca. 1 month | none | 20 | 38.5 | serum | N/A | N/A | - | - | - | - |
|  |  |  |  |  |  | OP fluid | + | - | 27.50 | 88.76 | + | 88.89 |
| 7735 | Serengeti, Tanzania | ca. 1 month | healed gum lesion | 19 | 39.0 | serum | N/A | N/A | - | - | - | - |
|  |  |  |  |  |  | OP fluid | N/A | N/A | - | - | - | - |
| 7737 | Serengeti, Tanzania | ca. 1 month | none | 21 | 38.4 | serum | N/A | N/A | - | - | - | - |
|  |  |  |  |  |  | OP fluid | N/A | N/A | 27.50 | 90.76 | - | 88.54 |
| 7739 | Serengeti, Tanzania | ca. 1 month | none | 20 | 38.0 | serum | N/A | N/A | - | - | - | - |
|  |  |  |  |  |  | OP fluid | N/A | N/A | 27.50 | 89.48 | - | - |
| 7741 | Serengeti, Tanzania | ca. 1 month | none | 20 | 38.8 | serum | N/A | N/A | - | - | - | - |
|  |  |  |  |  |  | OP fluid | N/A | N/A | - | - | - | - |
| 7742 | Serengeti, Tanzania | ca. 1 month | none | 24 | 38.1 | serum | N/A | N/A | - | - | - | - |
|  |  |  |  |  |  | OP fluid | N/A | N/A | - | - | - | - |
| 7645 | Serengeti, Tanzania | N/A | N/A | 20 | 39.6 | serum | N/A | N/A | - | - | - | - |
|  |  |  |  |  |  | OP fluid | N/A | N/A | - | - | - | - |
| 7648 | Serengeti, Tanzania | N/A | N/A | 20 | 38.5 | serum | N/A | N/A | - | - | - | - |
|  |  |  |  |  |  | OP fluid | N/A | N/A | - | - | - | - |
| 7649 | Serengeti, Tanzania | N/A | N/A | 22 | 38.6 | serum | N/A | N/A | - | - | - | - |
|  |  |  |  |  |  | OP fluid | N/A | N/A | 27.50 | - | - | - |
| 7650 | Serengeti, Tanzania | N/A | N/A | 21 | 36.6 | serum | N/A | N/A | - | - | - | - |
|  |  |  |  |  |  | OP fluid | N/A | N/A | - | - | - | - |
| 7601 | Serengeti, Tanzania | ca. 1 month | none | 25 | 36.4 | serum | N/A | N/A | - | - | - | - |
|  |  |  |  |  |  | OP fluid | N/A | N/A | - | - | - | - |
| 7602 | Serengeti, Tanzania | ca. 1 month | none | 26 | 36.9 | serum | N/A | N/A | - | - | - | - |
|  |  |  |  |  |  | OP fluid | + | N/A | 29.50 | 88.88 | - | - |
| 7603 | Serengeti, Tanzania | ca. 1 month | healed gum lesion | 22 | 37.4 | serum | N/A | N/A | - | - | - | - |
|  |  |  |  |  |  | OP fluid | N/A | N/A | 27.50 | 90.21 | - | - |
| 7607 | Serengeti, Tanzania | ca. 1 month | none | 20 | 36.8 | serum | N/A | N/A | - | - | - | - |
|  |  |  |  |  |  | OP fluid | + | 32 | 10.50 | 88.67 | + | 89.20 |
| 7609 | Serengeti, Tanzania | ca. 1 month | none | 23 | 36.0 | serum | N/A | N/A | - | - | - | - |
|  |  |  |  |  |  | OP fluid | - | N/A | 27.50 | 89.43 | - | - |
| 7610 | Serengeti, Tanzania | ca. 1 month | none | 24 | 37.1 | serum | N/A | N/A | - | - | - | - |
|  |  |  |  |  |  | OP fluid | - | 30 | 14.00 | 88.93 | + | 89.19 |
| 7615 | Serengeti, Tanzania | N/A | N/A | 26 | 36.3 | serum | N/A | N/A | - | - | - | - |
|  |  |  |  |  |  | OP fluid | N/A | N/A | - | - | - | - |
| 7625 | Serengeti, Tanzania | N/A | N/A | 20 | 37.6 | serum | N/A | N/A | - | - | - | - |
|  |  |  |  |  |  | OP fluid | N/A | N/A | - | - | - | - |
| Kcow1 | Nakuru, Kenya | N/A | none | N/A | 39.1 | serum | N/A | N/A | - | - | - | - |
| Kcow2 | Nakuru, Kenya | N/A | none | N/A | 39.0 | serum | N/A | N/A | - | - | - | - |
| Kcow3 | Nakuru, Kenya | N/A | none | N/A | 38.7 | serum | N/A | N/A | - | - | - | - |
| Kcow4 | Nakuru, Kenya | ca. 2 days | lesions, drop in milk production | N/A | 37.6 | serum | N/A | N/A | - | - | - | - |
|  |  |  |  |  |  | gum epi | - | N/A | 9.45 | 88.93 | + | 88.93 |
| Kcow5 | Nakuru, Kenya | N/A | none | N/A | 37.1 | serum | N/A | N/A | - | - | - | - |
| Kcow6 | Nakuru, Kenya | ca. 3-4 days | lesions, drop in milk production | N/A | 37.9 | serum | N/A | N/A | - | - | - | - |
| Kcow7 | Nakuru, Kenya | ca. 7-8 days | lesions | N/A | 37.0 | serum | N/A | N/A | - | - | - | - |
| Kcow8 | Nakuru, Kenya | ca. 3-4 days | lesions, drop in milk production | N/A | 37.8 | serum | N/A | N/A | 22.00 | 88.63 | - | - |
|  |  |  |  |  |  | OP fluid | N/A | N/A | - | - | - | - |
| Kcow9 | Nakuru, Kenya | ca. 1-2 days | lesions | N/A | 37.0 | serum | N/A | N/A | - | - | - | - |
|  |  |  |  |  |  | OP fluid | N/A | N/A | 12.75 | 88.99 | + | 88.99 |
|  |  |  |  |  |  | gum epi | - | N/A | 12.00 | 88.68 | + | 88.58 |
| Kcow10 | Nakuru, Kenya | ca. 1-2 days | lesions, drop in milk production | N/A | 37.4 | serum | N/A | N/A | 19.75 | 88.68 | - | - |
|  |  |  |  |  |  | OP fluid | N/A | N/A | 15.75 | 88.78 | + | 88.78 |
| Kcow11 | Nakuru, Kenya | N/A | none | N/A | 38.4 | serum | N/A | N/A | - | - | - | - |
